# RAD4 and RAD23/HMR Contribute to *Arabidopsis* UV Tolerance

**DOI:** 10.3390/genes9010008

**Published:** 2017-12-28

**Authors:** Triparna Lahari, Janelle Lazaro, Dana F. Schroeder

**Affiliations:** Department of Biological Sciences, University of Manitoba, Winnipeg, MB R3T 2N2, Canada; umlahari@myumanitoba.ca (T.L.); lazaroj4@myumanitoba.ca (J.L.)

**Keywords:** XPC/RAD4, RAD23, HEMERA, *Arabidopsis*, UV tolerance, nucleotide excision repair

## Abstract

In plants, exposure to solar ultraviolet (UV) light is unavoidable, resulting in DNA damage. Damaged DNA causes mutations, replication arrest, and cell death, thus efficient repair of the damaged DNA is essential. A light-independent DNA repair pathway called nucleotide excision repair (NER) is conserved throughout evolution. For example, the damaged DNA-binding protein Radiation sensitive 4 (Rad4) in *Saccharomyces cerevisiae* is homologous to the mammalian NER protein Xeroderma Pigmentosum complementation group C (XPC). In this study, we examined the role of the *Arabidopsis thaliana* Rad4/XPC homologue (AtRAD4) in plant UV tolerance by generating overexpression lines. AtRAD4 overexpression, both with and without an N-terminal yellow fluorescent protein (YFP) tag, resulted in increased UV tolerance. YFP-RAD4 localized to the nucleus, and UV treatment did not alter this localization. We also used yeast two-hybrid analysis to examine the interaction of AtRAD4 with *Arabidopsis* RAD23 and found that RAD4 interacted with RAD23B as well as with the structurally similar protein HEMERA (HMR). In addition, we found that *hmr* and *rad23* mutants exhibited increased UV sensitivity. Thus, our analysis suggests a role for RAD4 and RAD23/HMR in plant UV tolerance.

## 1. Introduction

All living organisms have an inherent ability to protect their genetic integrity against naturally occurring environmental mutagens. Ultraviolet (UV) light is one of the most common and unavoidable environmental sources of DNA damage. UV induces dipyrimidine photolesions in DNA, such as cyclobutane pyrimidine dimers (CPDs) and 6–4 photoproducts (6–4PPs) [1]. Damaged DNA arrests cellular processes such as replication and transcription. A failure to repair DNA damage ultimately leads to disturbances of gene expression, mutations, and apotosis. The mechanisms for the protection and repair of DNA are well-conserved [2]. The single-step light-dependent damage repair, called photoreactivation, is carried out by photolyase enzymes in the presence of blue light. Light-independent multistep DNA repair is called nucleotide excision repair (NER) [3]. Defective NER in humans can result in xeroderma pigmentosum (XP), Cockayne syndrome (CS), and trichothiodystrophy [4]. Xeroderma pigmentosum results in hypersensitivity to UV radiation and increased risk of skin cancer. Eight genetic complementation groups (A to G, V) have been identified for XP to date [5]. 

The initiation of NER varies depending on the location of the damage. The repair of transcribed DNA strands is known as transcription-coupled NER (TC-NER). Damage recognition in TC-NER is performed by Cockayne syndrome A and B (CSA and CSB) [6]. In contrast, global genomic NER (GG-NER) is the repair of untranscribed DNA across the entire genome [7]. In mammalian GG-NER, UV-induced lesions recruit the UV-damaged DNA-binding (DDB) protein complex, DDB1–DDB2–CUL4A–RBX1. The DDB complex then recruits the Xeroderma Pigmentosum complementation group C (XPC) complex to the damaged site. The mammalian XPC complex consists of XPC, hRAD23B, and Centrin 2 (CEN2), a small calcium-binding EF-hand protein. Damage recognition by the XPC complex is followed by damage verification by the basal transcription factor IIH (TFIIH) complex and XPA. During verification by TFIIH, two helicases, XPD and XPB, open the double helix with their ATPase activity. Replication protein A (RPA), along with the endonucleases XPG and XPF-ERCC1, become active after damage verification, resulting in a dual incision at the damaged site and the removal of the damage. Finally, repair synthesis completes the repair [8].

The yeast (*Saccharomyces cerevisiae*) XPC orthologue, Rad4 (radiation sensitive 4), has an activity that is similar to that of its mammalian counterpart. Rad4, along with yeast Rad23, forms a complex to recognize UV-damaged DNA. This complex is recruited by Rad7/Rad16. Rad4 is ubiquitinated and degraded after damage recognition [9].

The GG-NER process is similar in plants, mammals, and yeast [3]. Plant homologues of Rad23 have been reported in *Arabidopsis thaliana*, rice, and *Daucus carota* [10,11]. In *A. thaliana*, the RAD23 family includes four members, RAD23A, RAD23B, RAD23C, and RAD23D, which are also involved in the transfer of ubiquitinated proteins to the 26S proteasome [12]. In addition, Chen et al. [13] identified a novel protein in the *Arabidopsis* phytochrome nuclear body, HEMERA, previously known as pTAC12 [13,14]. HEMERA was found to be structurally similar to RAD23, and partially complements Rad23 function in yeast [13]. Studies involving the loss of *Arabidopsis* CEN2 (AtCEN2) confirmed its role in NER [15]. AtCEN2 interacts with the *Arabidopsis* homologue of human XPC (AtRAD4) via an EF-hand Ca^2+^-binding domain. This domain is required for CEN2 function in NER [16]. In plants, the role of AtRAD4 in DNA repair has yet to be studied in detail. In this study, we investigate the role of AtRAD4 in UV tolerance and its interaction with RAD23 and HMR. 

## 2. Materials and Methods 

### 2.1. Plant Material and Growth Conditions

In these studies, the Columbia-0 (Col-0) ecotype was used as the wild-type control. The *HEMERA* (At2g34640) partial loss-of-function allele *hmr-22* [17] was kindly provided by Dr. Meng Chen, UC Riverside. The lines *rad23a-1* (SALK_064980 in At1g16190), *rad23b-1* (SALK_076360 in At1g79650), *rad23c-1* (SALK_068091 in At3g02540), and *rad23d-1* (SALK_014137 in At5g38470) [12] were ordered from the Arabidopsis Biological Resource Center (ABRC), Columbus, OH, USA. Unfortunately, we were unable to identify homozygotes in the *rad23d-1* line, so *rad23a–c* were used for analysis. The seeds were sterilized (10 min in 70% ethanol, 0.5% Triton X-100 followed by 10 min in 95% ethanol) and plated on Linsmaier and Skoog (LS) medium (Caisson, Smithfield, UT, USA) containing 0.6% sucrose and 0.86% phytoblend (Caisson). The seeds were cold-stratified at 4 °C for 2 days, followed by growth in an incubator at 20 °C, 50% relative humidity, and long-day conditions for 14 days. Seedlings were transplanted to soil on the 14th day for further growth in long-day conditions (16 h light and 8 h dark) provided by fluorescent bulbs (100 μM photons m^−2^ s^−1^). The temperature was maintained at 20 °C and the relative humidity at 50%. The soil used for growth was Sunshine mix No. 1 (SunGro, Bellevue, WA, USA). 

### 2.2. Generation of Overexpression Lines 

Overexpression lines were generated using the *RAD4* (At5g16630) cDNA in the pENTR223 vector (G16664) obtained from the ABRC. The *RAD4* cDNA was cloned into pEarleyGate100 (CaMV 35S:RAD4) and pEarleyGate104 (CaMV 35S:YFP-RAD4) using the Gateway technology [18]. The pEarleyGate vectors were also obtained from the ABRC. Wild-type *Arabidopsis Col*-0 was transformed via agrobacterium-mediated transformation [19], and the transformed generation (T1) was screened with BASTA selection. Homozygous T3 plants were used for the experiments. 

### 2.3. RNA Extraction and qRT-PCR 

Col-0 and RAD4 overexpression lines were grown on LS plates in long-day conditions for 7 days. The RNA was extracted from 50 seedlings per sample using the RNeasy plant minikit (Qiagen, Hilden, Germany) including DNase treatment. cDNA was synthesized from RNA with the Maxima First Strand cDNA synthesis kit (Fermentas, Waltham, MA, USA). Transcript levels were checked with the *RAD4* cDNA specific primers RAD4_c671F (GTAAAGGCACAGCGGAAGAG) and RAD4_c780R (CCCAGGTTTTAAGGATGCAA). *EF1α* (At5g60390) (CTGGAGGTTTTGAGGCTGGTAT, CCAAGGGTGAAAGCAAGAAGA) was used to normalize the sample loading [20,21]. Real-time PCR was performed using SsoFast EvaGreen Supermix (Bio-Rad, Hercules, CA, USA), and the CFX Connect Real-time PCR detection system (Bio-Rad, Hercules, CA, USA) was used for analysis. Two biological replicates were performed per genotype, the data were normalized versus *EF1α*, and the mean values were calculated and expressed as relative to the levels in the Col-0 control. 

### 2.4. UV Sensitivity Assays

The seeds were sterilized and plated on LS medium containing 0.6% sucrose and 0.86% phytoblend. The plates were kept at 4 °C for 2 days for cold stratification, followed by vertical growth for 3 days at 20 °C in long-day conditions. The three-day-old plants were exposed to 0 or 1000 J m^−2^ UV-C (shortwave UV lamp XX-15S, UVP/LLC, Upland, CA, USA) and wrapped with aluminium foil to prevent photoreactivation. The plants were then rotated 90° and grown in the dark for 3 days. Plates were then scanned on a Perfection 1260 scanner (Epson, Suwa, Japan), and hypocotyl and root growth were measured for the indicated number of seedlings (n) using ImageJ. 

In the light-versus-dark UV sensitivity assay, the seeds were grown in the same conditions as above. Three-day-old seedlings were exposed to 0 or 1000 J m^−2^ UV-C, followed by 2 days growth in light or dark. The roots were measured and analyzed as above. 

In the adult assay, the seedlings were grown in the above conditions on LS medium and transplanted to soil at two weeks of age. At three weeks of age, the plants were exposed to 0, 300, or 600 J m^−2^ of UV-C, followed by 3 days of growth in dark conditions, then returned to long-day conditions. After 2–3 days of growth in the light, individual leaves were scored as either undamaged (green) or damaged (yellow or brown), and data were expressed as percentage of undamaged leaves (green leaves/total leaves) for six plants per genotype per treatment. In the adult UV sensitivity assays, *uvh1-1* was used as the positive control [22].

### 2.5. Protein Localization

Yellow fluorescent protein (YFP)-tagged RAD4 T3 and control seeds were sterilized and cold-stratified for 2 days at 4 °C. The seedlings were grown on LS medium for 3 days in the dark following a 6 h light treatment to initiate germination. The slides were prepared with 2–3 seedlings per slide in distilled water and were observed under an AXIO Imager Z1 Microscope (Zeiss, Oberkochen, Germany) equipped with Axio Vision 4.8 software, using YFP (Filter Set YFP-2427B-000, Semrock Inc., Rochester, NY, USA) and DAPI (Zeiss Filter Set 02 (488002-9901-000)) filters. DAPI (Sigma-Aldrich Canada, Oakville, ON, Canada) staining (10 μg/mL) [23] was done for 10 min. For UV treatments, the seedlings were exposed to 1000 J m^−2^ UV-C and then observed. 

### 2.6. Yeast Two-Hybrid Analysis

RAD4 protein interactions were investigated using the Matchmaker gold yeast two-hybrid system (Clontech, Mountian View, CA, USA). The cDNAs of *RAD4* (ABRC clone U16664), *RAD23B* (ABRC clone U09913), and *DDB2* (At5g58760) (ABRC clone U61992) were cloned into pGADT7 (Leu selection) and pGBKT7 (Trp selection) via standard cloning or Gateway technology. *RAD4* pGADT7 was digested with SacI enzyme to remove the C-terminal fragment of RAD4, then ligated to construct *RAD4N* pGADT7 (1-368/866 aa). To construct *RAD4N* pGBKT7 (1-371/866 aa), *RAD4* pGBKT7 was digested with PstI to remove the C-terminal end of RAD4, followed by re-ligation. *HEMERA* pGADT7 and pGBKT7 were kindly provided by Dr. Meng Chen, UC Riverside. Diploid yeast strains were plated via a series of five-fold dilutions on both double drop-out (–trp, –leu) control and quadruple drop-out (–ade, –his, –leu, –trp) selection medium along with the positive (p53/T) and negative (Lam/T) controls. Protein interaction resulted in the expression of *ADE2* and *HIS3* reporters and growth on the selective medium. On the control medium, Ade− colonies sometimes turn reddish over time while Ade+ colonies remain pale.

### 2.7. Statistical Analysis

A Student’s *t*-test was performed to compare the overexpression lines or mutants with Col-0 wild type. Values of *p* ≤ 0.05 were considered to be statistically significant. Each experiment was repeated at least twice, and representative data are shown.

## 3. Results

Initially, we attempted to examine the *AtRAD4* loss-of-function phenotype by examining two Salk T-DNA insertion lines (SALK_135310, SALK_020675) and one FLAG T-DNA line (FLAG_005E11) [24,25]. The T-DNA insertions in the Salk lines are beyond the 3′ end of the *RAD4* transcript, so they do not disrupt the coding sequence. The analysis of *RAD4* transcription in these two lines showed no decrease in *RAD4* levels (data not shown). Thus, these two lines are not suppressed or silenced and are not *RAD4* loss-of-function alleles. The insertion site of the FLAG T-DNA line is in exon 11. However, no homozygotes were found in the segregating populations, suggesting that *RAD4* loss of function results in gamete or embryo lethality.

**Figure 1:**
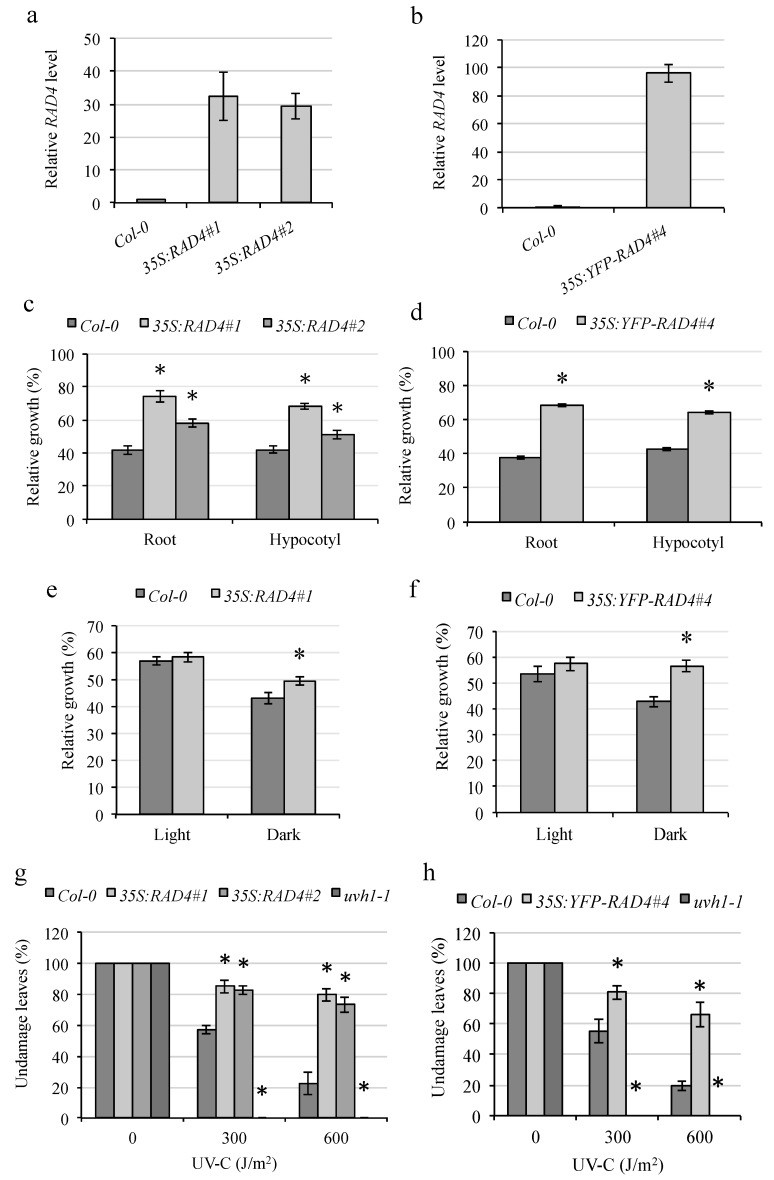
Overexpression of *Arabidopsis* Radiation sensitive 4 (RAD4) and yellow fluorescent protein (YFP)-RAD4 results in increased tolerance of ultraviolet (UV) radiation. *RAD4* levels in (**a**) 35S:RAD4 and (**b**) 35S:YFP-RAD4 overexpression lines, relative to control Col-0. The values are normalized relative to the reference gene *EF1α*. The error bars indicate SE (*n* = 2). (**c**–**h**) UV tolerance in 35S:RAD4 and 35S:YFP-RAD4 overexpression lines. (**c**,**d**) Hypocotyl and root growth in dark-incubated seedlings analyzed 3 days after UV treatment, expressed as relative to the untreated controls (*n* = 10). (**e**,**f**) Root growth in light- and dark-incubated seedlings analyzed 2 days after UV treatment, expressed as relative to the untreated controls (*n* = 10). (**g**,**h**) Percentage of undamaged leaves in adult plants after 0, 300, or 600 J m^−2^ UV treatment followed by dark incubation (*n* = 6). For c–h, the values are means ± SE, * = *p* < 0.05 for the overexpression lines versus Col-0 wild type in the same conditions.

Thus, in order to examine the role of AtRAD4 in UV tolerance, we utilized a gain-of-function approach by generating overexpression lines using pEarleyGate vectors [18]. Two overexpression (CaMV 35S driven) constructs were generated, one with an N-terminal YFP tag in the pEarleyGate104 vector, and one without a tag in the pEarleyGate100 vector. Both these constructs were transformed into the Col-0 wild-type background, transformed lines were generated, and overexpression was confirmed in the homozygous T3 lines (Figure 1a,b). The tagless-RAD4 overexpression lines showed increased UV tolerance in both the root and hypocotyl (Figure 1c). This increased UV tolerance occurred only when the plants were incubated in the dark following UV treatment, but not when they were incubated in the light (Figure 1e), consistent with a role in dark repair (nucleotide excision repair). In the adults, RAD4 overexpression resulted in decreased damage following UV treatment and dark incubation (Figure 1g and Figure S1), and, therefore, increased UV tolerance. Similarly, YFP-RAD4 overexpression also resulted in increased UV tolerance in dark-incubated hypocotyls, roots, and adult leaves (Figure 1d,h), but not in light-incubated roots (Figure 1f). Thus, RAD4 overexpression results in increased UV tolerance, and the N-terminal YFP tag does not appear to interfere with RAD4 function.

**Figure 2:**
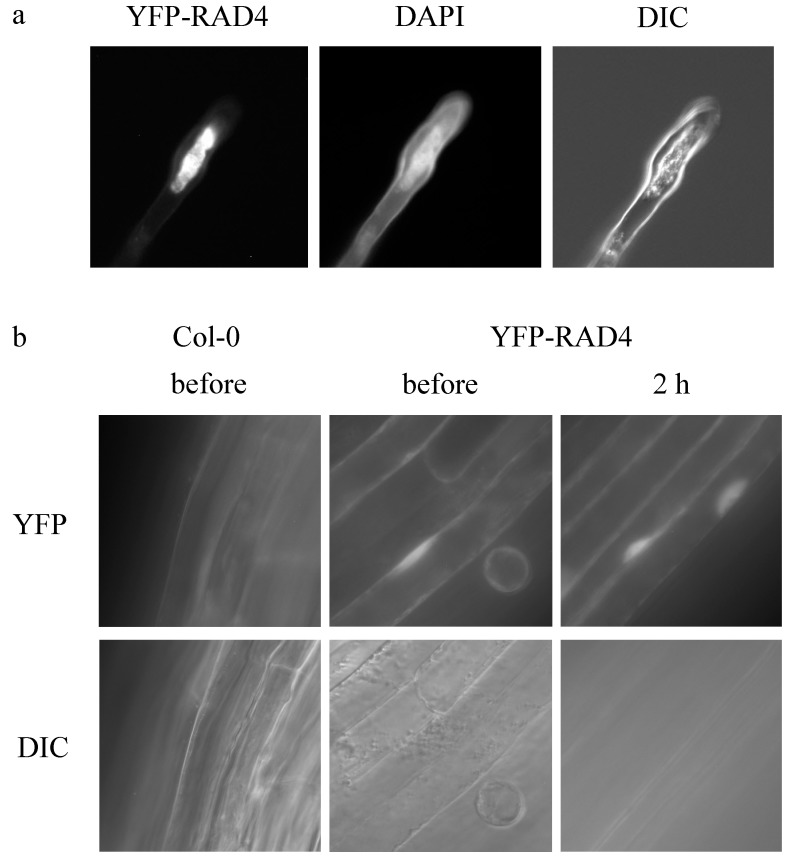
YFP-RAD4 exhibits nuclear localization. (**a**) Root hair cells in three-day-old 35S:YFP-RAD4 dark-grown seedlings were examined under 40× magnification using YFP fluorescence, DAPI staining, and differential interference contrast (DIC). (**b**) YFP-RAD4 in three-day-old hypocotyl cells observed under a YFP filter (top) and DIC (bottom), before and after UV treatment, under 40× magnification.

In order to determine RAD4 cellular localization, we examined YFP fluorescence in the YFP-RAD4 line. YFP-RAD4 was found to be nuclear-localized, as confirmed by DAPI staining (Figure 2a). While the Arabidopsis SubCellular Proteomic Database (SUBA) [26] predicts nuclear, plastid, and mitochrondrial localization for RAD4, both the consensus algorithms SUBAcon [27] and PSI (Plant Subcellular localization integrative predictor) [28] predict nuclear localization of AtRAD4, with scores of 0.998 and 0.708, respectively. AtRAD4 appeared to be localized throughout the nucleus, consistent with the prediction of the Sub-nuclear Compartments Prediction System (Version 2.0) [29], which predicts nucleoplasm and nuclear lamina localization for AtRAD4. This localization was not altered by UV treatment (Figure 2b). 

Yeast two-hybrid analysis was used to examine the interactions between the *Arabidopsis* GG-NER components. *Arabidopsis RAD4* and *DDB2* homologues were cloned into yeast two-hybrid vectors and tested for interaction, however no growth on the selective medium, indicative of interaction, was observed (Figure S2). In addition, RAD4 did not interact with itself in this assay (Figure S3).

**Figure 3:**
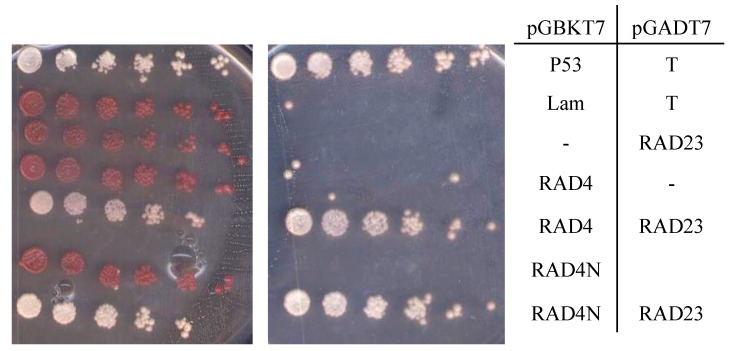
Yeast two-hybrid analysis of the interaction between the RAD4 N-terminus and RAD23B. The RAD4 N-terminal half is sufficient to interact with RAD23B. Five-fold dilutions of the indicated diploid strains were plated on the double drop-out (–leu, –trp) control plate on the left, and the quadruple drop-out (–leu, –trp, –ade, –his) selection medium on the right. The interaction between p53 and T, resulting in growth on the selective medium, is the positive control, whereas Lam and T, which do not interact, are the negative control.

We then tested for the interaction between the XPC complex components AtRAD4 and RAD23B. Interestingly, the RAD23B GAL4 DNA-binding domain fusion alone (RAD23 pGBKT7) resulted in growth even in the absence of RAD4 (Figure S4a), suggesting that RAD23B possesses some transcriptional activation activity. However, the RAD23 pGADT7 and RAD4 pGBKT7 constructs did not result in growth alone, but did together, indicating an interaction (Figure S4b). The N-terminal half (1-368/866 aa) of RAD4 was found to be sufficient for RAD23B interaction (Figure 3). 

**Figure 4:**
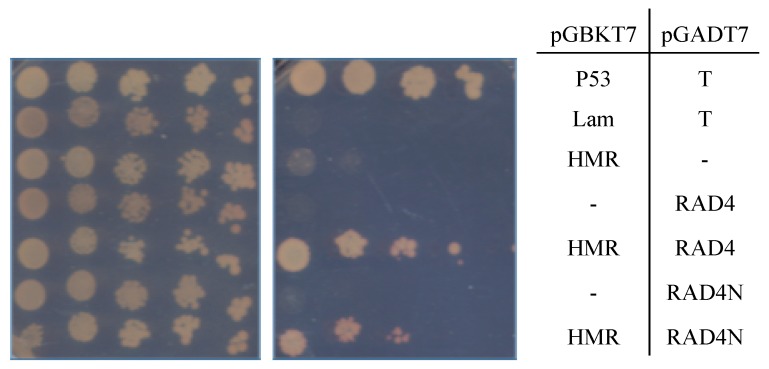
Yeast two-hybrid analysis of RAD4–HMR interaction. HEMERA (HMR) interacted with both full-length RAD4 and the RAD4 N-terminal half. Five-fold dilutions of the indicated diploid strains were plated on the double drop-out (–leu, –trp) control plate on the left, and the quadruple drop-out (–leu, –trp, –ade, –his) selection medium on the right. The interaction between p53 and T, resulting in growth on the selective medium, is the positive control, whereas Lam and T, which do not interact, are the negative control.

**Figure 5:**
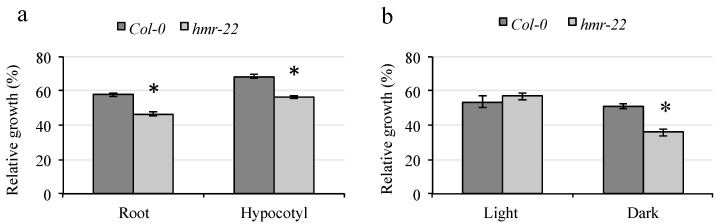
The mutant *hmr* exhibits increased UV sensitivity. (**a**) Hypocotyl and root growth in dark-incubated seedlings analyzed 3 days after UV treatment, expressed as relative to the untreated controls (*n* = 20). (**b**) Root growth in light- and dark-incubated seedlings analyzed 2 days after UV treatment, expressed as relative to the untreated controls (*n* = 20). The values are means ± SE, * = *p* < 0.05 for the mutant versus Col-0 wild type in the same conditions.

We also tested for the interaction between RAD4 and the RAD23-like protein HEMERA. Both RAD4 and the RAD4 N-terminal half (1-371/866 aa) were able to interact with HMR in yeast two-hybrid analysis (Figure 4). This result is consistent with the ability of HEMERA to partially rescue yeast *rad23* UV sensitivity [13]. To examine the role of HMR in *Arabidopsis* UV tolerance, we performed UV assays using a viable *hmr* loss-of-function allele, *hmr-22* [17]. The seedlings of *hmr-22* exhibited increased UV sensitivity in both roots and hypocotyls (Figure 5a), indicating that HMR contributes to *Arabidopsis* UV tolerance. This sensitivity was specific to dark conditions (Figure 5b).

**Figure 6:**
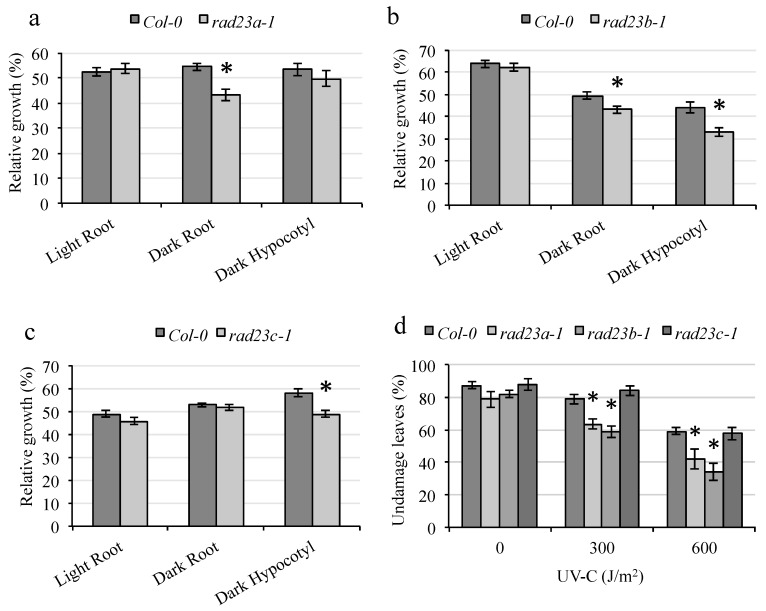
Mutants of *rad23* exhibit increased UV sensitivity. (**a**–**c**) Relative root and hypocotyl growth in light- or dark-incubated seedlings analyzed 2 days after UV treatment (*n* = 20). (**d**) Fraction of damaged leaves in adult plants after 0, 300, or 600 J m^−2^ UV treatment followed by dark incubation (*n* = 6). The values are means ± SE, * = *p* < 0.05 for the mutant versus Col-0 wild type in the same conditions.

Since the RAD23-like protein HMR contributes to UV tolerance, we examined UV sensitivity in *rad23a*, *rad23b*, and *rad23c* mutants. The mutant *rad23a* exhibited sensitivity in dark-grown roots (Figure 6a), while *rad23b* was sensitive in both dark-grown roots and hypocotyls (Figure 6b). The mutant *rad23c* was sensitive in dark-grown hypocotyls only (Figure 6c). In adult plants, *rad23a* and *rad23b* exhibited increased leaf damage and, therefore, increased sensitivity (Figure 6d). Thus, despite a potential redundancy within the RAD23 family, *rad23* single mutants were sensitive to UV damage.

## 4. Discussion

The DNA damage recognition factor XPC/RAD4 has been well studied in both mammalian [30] and yeast systems [31] with respect to its role in the repair of UV-damaged DNA. In this study, we examined the role of *Arabidopsis* RAD4 in UV tolerance using a gain-of-function strategy. AtRAD4 overexpression lines, both with and without YFP tags, resulted in a significant increase in UV tolerance in hypocotyls, roots, and adults, indicating that increased AtRAD4 results in increased UV tolerance. This result suggests that RAD4 is limiting during *Arabidopsis* nucleotide excision repair of UV-damaged DNA, and is the first genetic evidence that RAD4 contributes to this process in plants.

Our results are in contrast to findings in yeast, where ScRad4 overexpression in the wild type did not result in increased UV tolerance. However, ScRad4 overexpression did result in partial rescue of UV sensitivity in *rad23* mutants, consistent with the proposed role of Rad23 in Rad4 stabilization [32]. Human *XPC* was found to be overexpressed in hepatocellular carcinoma cells, and may contribute to chemotherapeutic resistance in these cells [33]. The role of XPC overexpression was also studied with respect to p53 turnover in the presence and absence of UV. In the absence of UV, XPC overexpression did not affect p53 turnover. However, in the presence of UV, XPC overexpression resulted in increased p53 degradation, indicating a role for XPC in p53 turnover [34]. Thus, the effect of XPC/RAD4 overexpression varies between systems. 

The overexpression of other *Arabidopsis* NER components, for example DDB2 and DDB1a, was also shown to increase UV tolerance [35,36]. Extracts of plants overexpressing AtCEN2 exhibited increased repair of UV-damaged DNA [16]. In contrast, the overexpression of the transcription-coupled repair component AtCSA resulted in decreased UV tolerance [37]. 

The dark-specific UV tolerant phenotype in RAD4-overexpression lines is consistent with AtRAD4 acting during nucleotide excision repair. To determine if RAD4 exhibited nuclear localization, consistent with this role, we examined AtRAD4 cellular localization and found it to be nuclear-localized. UV treatment did not affect this pattern. In general, our localization results are in agreement with those generated by in silico localization prediction analysis [26,27,28,29].

In yeast, ScRad4 was found to be localized in both the cytoplasm and the nucleus in a GFP-tagged localization study of yeast proteins [38]. Subsequently, the response of ScRAD4 to DNA damaging agents was studied. ScRAD4 was found throughout the cell in control conditions and localized in the nucleus after treatment with a DNA-damaging agent [39]. Mammalian XPC was observed in the nucleus using immunofluorescence [40]. Although XPC is localized in the nucleus, the localization is not homogenous. The heterogeneous localization of XPC was higher in areas of condensed chromatin [41]. XPC was found to become enriched in the perichromatin region following UV irradiation [42]. 

The localization of other *Arabidopsis* NER components was also examined using fluorescent tags. Like AtRAD4, DDB2 is localized throughout the nucleus [36,37]. Initial studies in onion cells indicated that CUL4 and DDB1a were nuclear-localized, while DDB1b localized to both the nucleus and the cytoplasm [43]. However, in studies of transgenic *Arabidopsis* lines, GFP-tagged DDB1a was observed in the cytoplasm, but shuttled to the nucleus after UV irradiation. The DNA damage response component ATR1 was required for DDB1a relocalization [36]. In another study, the XPC complex component AtCEN2 was observed in the cytoplasm in control conditions, but localized to the nucleus following UV irradiation [16]. The transcription-coupled repair protein CSA exhibited a speckled pattern in the nucleus [37], while the rice TFIIH component OsREX1 was also nuclear [44]. Since NER occurs in the nucleus following UV damage, it appears that NER components already in the nucleus remain in place, while those present in the cytoplasm in control conditions relocate to the nucleus in response to UV treatment. 

We examined RAD4 interaction with other NER components using yeast two-hybrid assays. We did not detect an interaction between AtRAD4 and the damage recognition factor DDB2 in this assay. The mammalian XPC was found to interact with DDB2, however this was not detected using yeast two-hybrid analysis but via coimmunoprecipitation [45]. Other proteins, such as DDB1, would have been present in this case, suggesting that the cellular context of the DDB and XPC complexes, or perhaps in vivo post-translational modifications, are required for DDB2–XPC/RAD4 interaction. 

We did detect an interaction between AtRAD4 and RAD23b via yeast two-hybrid analysis and showed the RAD4 N-terminal half was sufficient for RAD23b interaction. This region (amino acid 1–368/866) corresponds to amino acid 1–270/755 in ScRad4 and 1–490/940 in mammalian XPC [31]. In yeast, ScRad4 was also found to interact with Rad23 in yeast two-hybrid analysis [46]. Interestingly, den Dulk et al. [47] found that the equivalent N-terminal ScRAD4 fragment did not interact with Rad23 in yeast two-hybrid analysis, but the C-terminal half did. However, cocrystallization of yeast Rad4 and Rad23 revealed contacts between the two proteins in both the N- and C-terminus of Rad4 [31]. In mammals, the C-terminal 160 amino acids of XPC were found to be essential for hRAD23a/b interaction in yeast two-hybrid analysis [48]. In vitro pulldown experiments found two XPC regions, 495–606 and 606–734, that were necessary but not sufficient for hRAD23b interaction, however the 495–734 region was sufficient [49].

We also found that AtRAD4 interacts with the RAD23-like protein HEMERA in yeast two-hybrid assays. Again, the RAD4 N-terminal half was sufficient for this interaction. HMR was previously found to partially rescue UV sensitivity in yeast *rad23* mutants, where it presumably would interact with ScRAD4 [13]. A C-terminally tagged HMR-CFP fusion protein was found to be localized to the chloroplast, while the N-terminally tagged YFP-HMR was found to be localized in the nucleus and cytoplasm but not in the chloroplast [13]. Thus, AtRAD4 and HMR could potentially interact in the nucleus, however this has yet to be assessed. HMR was shown to contribute to transcriptional activation [17,50]. We found that RAD23b was also able to activate transcription in yeast two-hybrid assays.

Since RAD4 interacted with HMR and RAD23b, we examined the role of HMR and the RAD23 family in *Arabidopsis* UV tolerance. We found the *hmr* and *rad23* mutants to be UV sensitive. This is somewhat surprising given that there are four RAD23 homologues in *Arabidopsis* that were found to act redundantly with respect to developmental phenotypes [12]. However, the *rad23d* mutant was previously found to exhibit UV-sensitive pollen development [51]. We found that *hmr* and *rad23a,b,c* all exhibit dark-specific UV sensitivity, consistent with roles for both HMR and the RAD23s in *Arabidopsis* nucleotide excision repair.

## 5. Conclusions

Our studies have shown that AtRAD4 overexpression results in increased UV tolerance, and that RAD23B and HMR interact with RAD4 and contribute to *Arabidopsis* UV tolerance. These results are consistent with a role for AtRAD4, RAD23, and HMR in plant GG-NER. These studies provide additional insights into the basis of plant DNA repair and provide the basis for potential future improvements of plant UV tolerance.

## Supplementary Materials

The following are available online at www.mdpi.com/2073-4425/9/1/8/s1. Figure S1: RAD4 overexpression increases adult UV tolerance. Figure S2: Yeast two-hybrid analysis of RAD4–DDB2 interaction. Figure S3: Yeast two-hybrid analysis of RAD4 self-interaction. Figure S4: Yeast two-hybrid analysis of RAD4–RAD23B interaction.
