# Supplementary material for: RAD4 and RAD23/HMR Contribute to Arabidopsis UV Tolerance
Source: Genes (Basel). 2017 Dec 28;9(1):8. doi: 10.3390/genes9010008 (PMC5793161; doi:10.3390/genes9010008)
Supplement: Supplementary file 1 [file genes-09-00008-s001.pdf]

RAD4 and RAD23/HMR contribute to *Arabidopsis* UV tolerance

Triparna Lahari, Janelle Lazaro and Dana F. Schroeder

Department of Biological Sciences, University of Manitoba, Winnipeg, MB R3T 2N2

Dana.Schroeder@umanitoba.ca

Supplemental material

0 J 300 J 600 J

Col-0 35S:RAD4#2 Col-0 35S:RAD4#1 Col-0 35S:RAD4#1

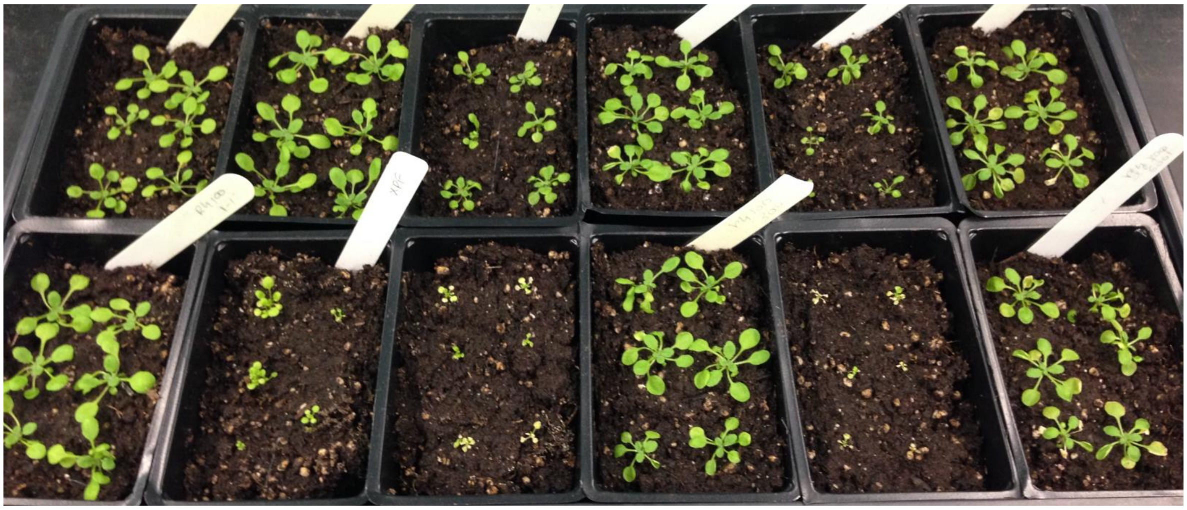

35S:RAD4#1 *uvh1-1* *uvh1-1* 35S:RAD4#2 *uvh1-1* 35S:RAD4#2

Supplemental Figure S1. RAD4 overexpression increases adult UV tolerance

21-day-old plants were treated with 0, 300, or 600 J m^{-2} UV-C followed by 3 days dark incubation and 3 days in long-day conditions.

a

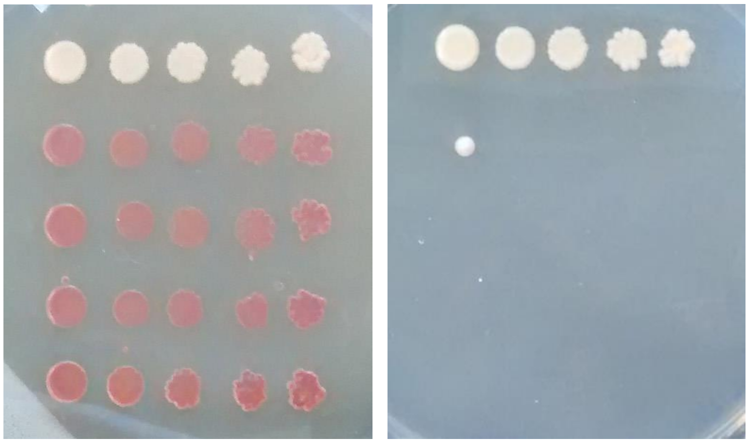

pGBKT7	pGADT7
P53	T
Lam	T
DDB2	-
-	RAD4
DDB2	RAD4

b

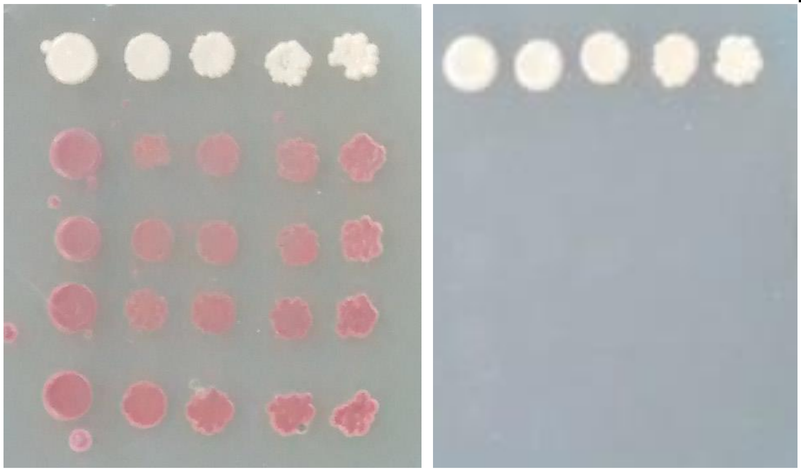

pGBKT7	pGADT7
P53	T
Lam	T
RAD4	-
-	DDB2
RAD4	DDB2

Supplemental Figure S2. Yeast two-hybrid analysis of RAD4-DDB2 interaction.

a) No interaction was observed between DDB2 pGBKT7 and RAD4 pGADT7. b) No interaction was observed between RAD4 pGBKT7 and DDB2 pGADT7. Five fold dilutions of the indicated diploid strains were plated on the double drop-out (-leu -trp) control plate on the left, and the quadruple drop-out (-leu –trp –ade -his) selection medium on the right. The interaction between p53 and T, resulting in growth on the selective medium, is the positive control, whereas Lam and T, which do not interact, are the negative control.

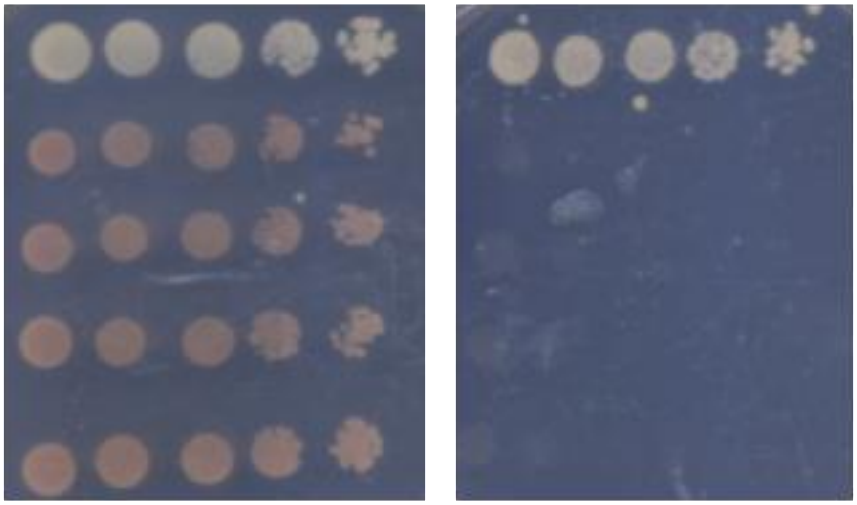

pGBKT7	pGADT7
P53	T
Lam	T
-	RAD4
RAD4	-
RAD4	RAD4

Supplemental Figure S3. Yeast two-hybrid analysis of RAD4 self-interaction.

No interaction was observed between RAD4 pGBKT7 and RAD4 pGADT7. Five fold dilutions of the indicated strains were plated on the double drop-out (-leu -trp) control plate on the left, and the quadruple drop-out (-leu -trp –ade -his) selective medium on the right. The interaction between p53 and T, resulting in growth on the selective medium, is the positive control, whereas Lam and T, which do not interact, are the negative control.

a

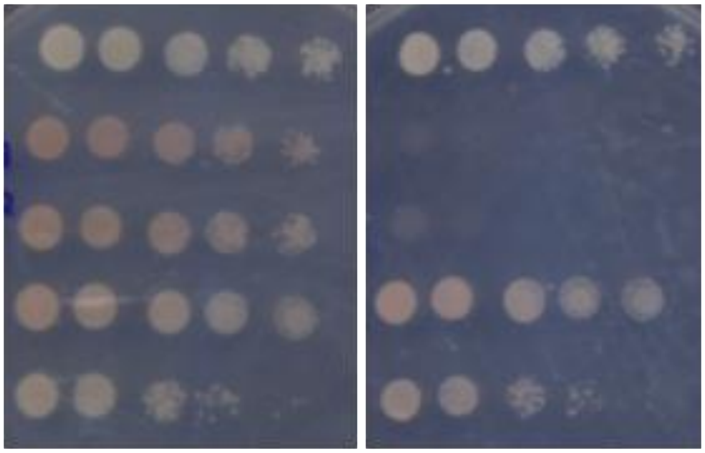

pGBKT7	pGADT7
P53	T
Lam	T
-	RAD4
RAD23B	-
RAD23B	RAD4

b

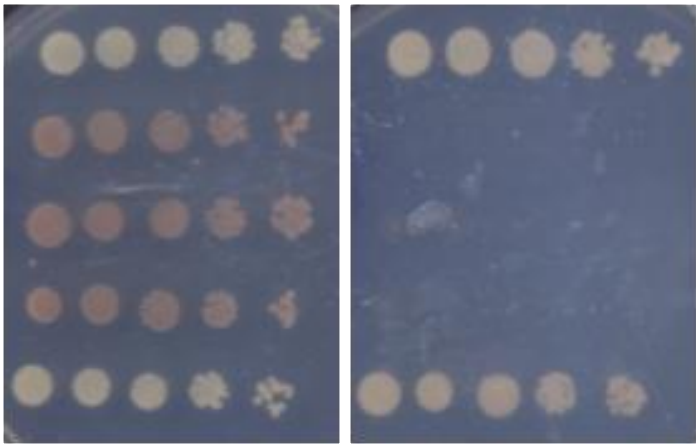

pGBKT7	pGADT7
P53	T
Lam	T
RAD4	-
-	RAD23B
RAD4	RAD23B

Supplemental Figure S4. Yeast two-hybrid analysis of RAD4-RAD23B interaction.

a) RAD23B pGBKT7 exhibited growth on selective medium with both empty pGADT7 and RAD4 pGADT7. b) Interaction was detected between RAD4 pGBKT7 and RAD23B. Five fold dilutions of the indicated strains were plated on the double drop-out (-leu -trp) control plate on the left, and the quadruple drop-out (-leu-trp-ade-his) selective medium on the right. The interaction between p53 and T, resulting in growth on the selective medium, is the positive control, whereas Lam and T, which do not interact, are the negative control.
